# Temporal Changes in Subcutaneous Fibrosis in Patients with Lower Extremity Lymphedema Following Surgery for Gynecologic Cancer: A Computed Tomography-Based Quantitative Analysis

**DOI:** 10.3390/diagnostics12081949

**Published:** 2022-08-12

**Authors:** Soyoung Lee, Dong Gyu Lee, Kyoung Tae Kim

**Affiliations:** 1 Department of Rehabilitation Medicine, Keimyung University Dongsan Hospital, Keimyung University School of Medicine, Daegu 42601, Korea; 2 Department of Physical Medicine and Rehabilitation, Yeungnam University College of Medicine, Daegu 42415, Korea

**Keywords:** lymphedema, computed tomography, fibrosis, inflammation, quantitative analysis

## Abstract

Lymphedema causes inflammation, which provokes fibrosis within the epifascial tissue. Temporal change in fibrosis according to severity of the lymphedema has not been widely investigated. We aimed to study the quantitative changes in epifascial fibrosis during lymphedema treatment using computed tomography (CT). Forty-five patients (mean age: 57.75 ± 11.12 years) who developed lymphedema following gynecologic surgery were included in this retrospective study. Two weeks of complete decongestive therapy and continued self-bandaging or compression garments were prescribed under regular follow-up monitoring. Lower-extremity epifascial fibrosis was quantitatively analyzed on the initial and follow-up CT scans. Circumference, skin fibrosis, subcutaneous tissue, and fibrosis ratio were calculated in the axial scan. Based on the change in lymphedema severity, we divided subjects into ‘improved’ and ‘aggravated’ groups. The affected lower extremities showed higher circumference, more skin fibrosis and subcutaneous tissue, and higher fibrosis ratio than the unaffected sides on initial CT scan. At follow-up, compared to the aggravated group, the improved group showed significant decreases in fibrosis of skin and subcutaneous tissue and fibrosis ratio. Subcutaneous fibrosis was reversible with volume resolution of lymphedema. Therapeutic approaches should be established on the basis of the reversible nature of fibrotic changes in patients with lower extremity lymphedema.

## 1. Introduction

Lymphedema is a burden on survivors of gynecologic cancer. Lymph node dissection is performed for cancer staging and to prevent metastasis [1]. An external iliac lymph node is a key regional lymph node draining lymphatic fluids from the lower extremities and pelvic cavity organs. Therefore, lymph node dissection (LND) breaks down the lymphatic vascular network, resulting in lymphedema. Bypassing deep lymph circulation and superficial lymph collecting vessels can occasionally replace regional deep lymph node function in the upper extremities [2]. However, these compensatory circulation systems are not effective in the pelvic region for lymphatic drainage from the lower extremities. Patients with lower extremity lymphedema experience more severe symptoms and complications than those with upper extremity lymphedema [3].

Occasionally, there is a time interval between surgical LND and development of lymphedema [4]. Inflammation can be one of the reasons for the delayed development of lymphedema [5]. Lymphatic fluid is protein-rich and possesses immunological properties which trigger the inflammatory mechanism. Inflammation stimulates fibroblasts to produce collagen fibers, the accumulation of which results in fibrosis [6]. This fibrous tissue is tough and not as elastic as the parent soft tissue. Therefore, it interferes with the natural functions of the parent tissue. The fibrous tissue decreases the collecting function of lymphatic capillaries [7]. Additionally, fibrosed lymph vessels lose contractile properties to propel lymph fluid [8]. Reduced lymphatic drainage from the tissue results in stasis of lymphatic fluid within subcutaneous tissues, causing lymphedema.

The resolution of fibrosis and inflammation depends on the characteristics of the tissues [9]. In highly regenerative liver and skin tissues, fibrosis is reversible. However, in intervertebral discs, tendons, and ligaments, fibrosis persists as scar tissue. Previous research has shown fibrotic changes within subcutaneous tissue following lymphedema [10,11]. However, no research has been conducted to elucidate the recovery of subcutaneous fibrosis following lymphedema treatment. If subcutaneous fibrosis following lymphedema can be shown to be modified by treatment, this will be promising evidence to advocate for the treatment of lymphedema.

We previously reported that subcutaneous fibrosis correlates with severity of lymphedema [12]. Therefore, this study aimed to investigate whether subcutaneous fibrosis resulting from lower-extremity lymphedema following pelvic lymphadenectomy is reversible with lymphedema treatment.

## 2. Materials and Methods

### 2.1. Subjects

We investigated patients with lower-extremity lymphedema following pelvic lymphadenectomy who were admitted to our rehabilitation department between January 2007 and April 2020. Data were collected retrospectively from hospital records. The inclusion criteria were as follows: (1) patient diagnosed with gynecologic cancer who underwent pelvic lymphadenectomy, (2) unilateral lower-extremity lymphedema diagnosed on the basis of clinical presentation and lymphoscintigraphy, (3) follow-up lower-extremity computed tomography (CT), and (4) received complete decongestive therapy and continued self-bandaging or compression garments. The exclusion criteria were as follows: (1) bilateral lower extremity lymphedema, (2) vascular or other systemic diseases causing lower extremity edema, and (3) absence of follow-up CT scans. In patients with gynecological malignancies, cancer cells might invade both the pelvic and paraaortic lymph nodes; thus, surgery or radiation therapy is often performed on both sides for radical treatment. In order to clarify the difference between the affected and unaffected limbs, unilateral lymphedema was defined as the absence of radioactive uptake in the pelvic lymph nodes of the affected limb on lymphoscintigraphy.

This study was approved by the Institutional Review Board (2021-06-084) and a request to waive the informed consent statement was approved.

### 2.2. Lymphedema Management

All patients received complete decongestive therapy (CDT), including short-stretch compressive bandaging, massage, and pneumatic compression for two weeks on admission and were trained in the self-bandaging method. After discharge, all patients were instructed to apply daily compressive self-bandages for over 20 h a day. If patient compliance was very poor, or if bandaging was impossible because of adverse effects of the bandage, alternative compressive garments were used. Regular outpatient visits were conducted to monitor patient self-management and encourage treatment compliance.

CT scans were performed in the initial period before CDT and at follow-up, approximately two years after the initial CT scan. To evaluate changes in fibrosis of the subcutaneous tissue according to severity of lymphedema, we classified the subjects into two groups: improved and aggravated, on the basis of the circumference difference between the initial and follow-up CT scans at each level. The improved and aggravated groups showed decreased and increased circumferences on subsequent CT scans, respectively.

### 2.3. CT-Based Quantitative Measurement of Lymphedema

All included patients underwent repeated lower-extremity CT scans (SOMTOM FORCE; Siemens, Erlangen, Germany). We selected four representative levels of CT scan images for the circumference and volume of fibrosis analysis. Axial images of U20 (20 cm above the upper margin of the patellar bone), U10 (10 cm above the upper margin of the patellar bone), L10 (10 cm below the lower margin of the patellar bone), and L20 (20 cm below the lower margin of the patellar bone) were obtained.

We used FIJI software (http://fiji.sc/Fiji (accessed on 2 March 2022)) for quantitative analysis [13]. FIJI is a distribution of the popular open-source software ImageJ. Semi-automated methods using FIJI software to measure limb circumference and subcutaneous fibrosis were performed.

The compartment of the lower extremity is composed of skin, subcutaneous tissue, muscle, and bone. Each territory has its own Hounsfield Units (HU) on CT scan, and it is possible to evaluate each territory quantitatively using the FIJI software [14]. Once the axial CT image is obtained, the corresponding region can be visually distinguished after setting the specific HU threshold, and semi-automated calculation of selected regions is possible. Details of the analysis protocol have been previously described [12].

Circumference, cross-sectional area (CSA) of the skin, and subcutaneous tissue fibrosis were measured to evaluate lymphedema severity. Fibrosis ratio was also evaluated to estimate the degree of fibrosis. The fibrosis ratio was calculated as follows:

(1) $$\mathrm{Fibrosis}\;\mathrm{ratio}\;=\frac{\mathrm{Fibrosis}\;\mathrm{of}\;\mathrm{skin}\;\mathrm{with}\;\mathrm{subcutaneous}\;\mathrm{tissue}\;\mathrm{area}\;}{\;\mathrm{Skin}\;\mathrm{with}\;\mathrm{subcutaneous}\;\mathrm{tissue}\;\mathrm{area}\;}\times 100$$

### 2.4. Statistics

All parameters were calculated for both the initial and follow-up CT scans. Wilcoxon signed-rank tests were used to compare parameters between the affected and unaffected sides on the initial CT scans. We compared parameters between initial and follow-up CT scans to evaluate whether change in lymphedema severity can modify fibrosis. The differences in circumference and fibrosis of the subcutaneous tissue and skin between the affected and unaffected sides were outcomes for statistical analysis. However, only the fibrosis ratio on the affected side was used in statistical analysis. We used the Mann–Whitney U test to compare parameters between the improved and aggravated groups. Statistical analyses were performed using IBM SPSS Statistics for Windows, Version 25.0 (IBM Corp., Armonk, NY, USA). Statistical significance was set at *p* < 0.05.

## 3. Results

### 3.1. Patient Characteristics

Forty-five patients (mean age: 57.75 ± 11.12 years) with lymphedema following pelvic lymphadenectomy were included in this study (Table 1).

### 3.2. Initial CT Scan

The mean duration (months) between the initial CT scan and surgery was 54.73 ± 61.15. At the initial CT scan, the parameters on the affected side were significantly higher than those on the unaffected side (Table 2) due to subcutaneous fibrosis.

### 3.3. Fibrotic Changes

**Figure 1:**
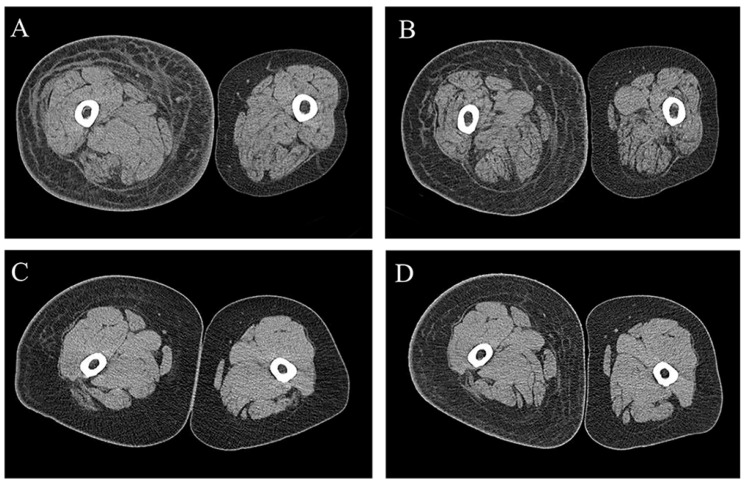
Bidirectional change of subcutaneous fibrosis in computed tomography (CT) scan. Fibrosis was improved in follow-up (**B**) than initial (**A**). Fibrosis was aggravated in follow-up (**D**) than initial (**C**).

**Figure 2:**
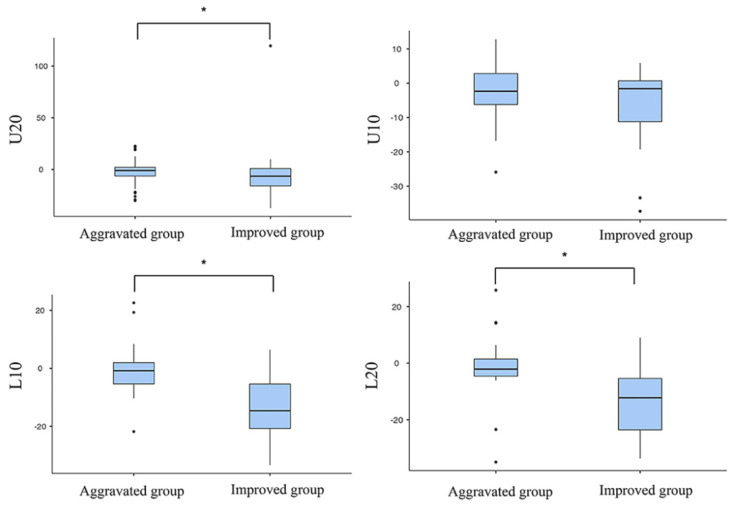
The fibrosis ratio at each level of the lower extremities. The fibrosis ratio within affected side between initial and follow-up CT scans was significantly different between the improved and aggravated groups at U20, L10, and L20. CT, Computed tomography; L10, 10 cm below the lower margin of the patellar bone; L20, 20 cm below the lower margin of the patellar bone; U10, 10 cm above the upper margin of the patellar bone; U20, 20 cm above the upper margin of the patellar bone. * *p* < 0.05.

The fibrotic changes showed bidirectional alterations. Fibrosis of skin and subcutaneous tissue showed a tendency to decrease in the improved group with reduced circumference; however, in the aggravated group, an opposite trend was observed (Figure 1). The improved group showed a significant decrease in fibrosis of the skin and subcutaneous tissue compared with the aggravated group at the U10 and L20 levels (Table 3). The fibrosis ratio of the improved group was also significantly lower than that of the aggravated group at the U20, L10, and L20 levels (Table 3, Figure 2).

## 4. Discussion

We aimed to study the quantitative changes in epifascial fibrosis during lymphedema treatment, using CT. Lower extremity lymphedema following pelvic lymphadenectomy stimulates subcutaneous fibrosis. When subcutaneous tissue fibrosis was quantitatively analyzed using CT images, there was a strong correlation with circumference, representing the severity of lymphedema. The results showed that the degree of fibrosis decreased if the severity of lymphedema ameliorated. Therefore, the degree of subcutaneous fibrosis following lymphedema showed bidirectional alterations according to change in lymphedema severity.

Inflammation is the entire local reaction in vascularized tissues with a final objective of repair and healing of the injured tissue sections. Tissue injuries generate polypeptides and lipopolysaccharides, which induce an immune reaction [15]. Although inflammation culminates in regeneration and repair, the initial phases cause deterioration of tissue architecture and function [16]. Subsequently, stimulated immune cells promote fibrosis by recruiting fibroblasts at the site, which is a scarring process that accumulates collagen fibers and extracellular matrix (ECM). Fibroblasts have a dual action of recruiting lymphocytes, by releasing chemokines during acute inflammation, and maturing into fibrosis in chronic inflammation [17]. Therefore, inflammation, caused by tissue injury, is the initiating event that progresses into fibrosis by inducing collagen synthesis [18].

Lymphedema causes inflammation within the subcutaneous areas of the affected extremities. Stasis of protein-rich lymphatic fluid within the subcutaneous area can trigger an immune reaction [5]. Chronic inflammation following lymphedema replaces functional tissues with collagen fibers and ECM, thereby decreasing interstitial lymphatic drainage.

As a result of chronic inflammation, patients with lymphedema exhibit skin and subcutaneous thickness and stiffness. Several modalities have been introduced to measure skin stiffness and thickness in lymphedema [19]. Ultrasonography is an easily accessible modality to evaluate soft tissue. In particular, shear wave elastography (SWE) was introduced to assess soft tissue stiffness quantitatively [20]. The elasticity of tissues was measured by the shear wave propagation speed to the adjacent tissues. The velocity is high in stiff tissues. SWE showed high density and thickness within skin following upper extremity lymphedema [21]. According to the lower extremity lymphedema stage, SWE shows higher skin stiffness on the affected than unaffected sides [22]. However, lower extremities have a high volume and larger area than upper extremities. Therefore, evaluating subcutaneous fibrosis of the lower extremities using ultrasonography has limitations. Three-dimensional CT scan provides a better analysis of large tissue compared to ultrasonography. Moreover, a CT scan can quantify tissues using HU. There is a lack of research on CT scan analysis of subcutaneous fibrosis [23,24]. We evaluated epifascial fibrosis using the open analysis tools FIJI software, which is convenient for clinicians [12].

In most cases, secondary lymphedema arises from lymph node dissection performed for preventing metastasis and recurrence of gynecological malignancies, including cervical, endometrial, ovarian, and vulvar cancers [25,26]. The procedure disrupts the lymphatic circulation and causes lymph stasis and lymphedema.

In cancer survivors, a time interval is observed between lymph node dissection and the development of lymphedema [27]. Although dissection of the lymph nodes causes delay or blockage of the drainage of lymphatic fluid, inflammation caused by lymph stasis aggravates lymphedema. Therefore, modulating inflammation following lymph node dissection is a promising treatment strategy. Pilot studies have evaluated the effectiveness of anti-inflammatory drugs for lymphedema [28,29]. Non-steroidal anti-inflammatory drugs (NSAIDs) and anti-Th2 immunotherapy for lymphedema reduce skin hyperkeratosis, thickness, and fibrosis. Therefore, reducing or modulating inflammation may help to resolve lymphedema.

The progression from lymph stasis to fibrosis can be inhibited in the early stage by removing the edema and activating the circulation of retained lymph fluid, which drives the inflammatory process [30]. Clinically, massage, bandaging, and compressive garments have been widely used to treat lymphedema [31]. However, fibrotic changes have been known to be irreversible after stage II lymphedema [32]. Recently, various surgical approaches have been attempted to prevent or reduce the severity of lymphedema [33]. A high degree of heterogeneity in the results was observed, depending on the surgical technique, patient selection, and the stage of lymphedema. However, most studies reported significantly reduced lymphedema volume with liposuction, lymphovenous bypass, and vascularized lymph node transfer compared to compression therapy only [34,35,36]. Although there is ample evidence of the positive impact of surgical treatment, there is a lack of research on the consequential change in fibrosis in the subcutaneous tissue. We found no studies on quantitative correlation between degree of fibrosis and type of lymphatic surgery.

Our study showed that reduced lymphedema strongly correlated with a decrease in the fibrosis caused by inflammation. Because the absolute volume of fibrosis is inevitably affected by the weight or height of the patient, we used the fibrosis ratio to evaluate the degree of change in fibrosis more accurately. The fibrosis ratio was lower at all levels in the improved group, and it was significantly lower than that in the aggravated group at the U20, L10, and L20 levels. The reason for the different result at the U10 level is unclear, but we expect similar trends when the sample size increases.

As this was a retrospective study, the type of lymphedema treatment after discharge, and differences in adherence to treatment among patients were difficult to quantify. Moreover, the duration from the first symptom onset to the initial CT scan and the time interval for the follow-up CT scan differed significantly among patients. Therefore, the results are insufficient to conclude which type or amount of lymphedema treatment can reduce fibrosis. However, the results suggest that fibrosis caused by lymphedema is reversible and is likely to be improved by treatment of the lymphedema.

This study has some limitations. First, we did not assess the type, duration, and adherence to compressive bandage treatment during follow-up, which could influence the effectiveness of lymphedema treatment [37]. Self-bandaging using a short stretch bandage is more challenging and the compliance rate is low [38,39]. Therefore, a prospective study is required to evaluate the relationship between compliance to treatment and impact on fibrosis. Second, there is no evidence to support whether reduction of inflammation precedes or follows the improvement of lymphedema—whichever happens first is assumed to be important for fibrosis. While we could evaluate the correlation between changes in skin and subcutaneous fibrosis and changes in lymphedema severity, measuring the degree of inflammation in the presence of lymphedema is challenging. Third, the stage of lymphedema was not considered, and the degree of change in fibrosis according to the symptom severity could not be measured. Fourth, the incidence of infection during the follow-up CT interval was not considered. The onset of lymphangitis not only increases edema abruptly but is also an important factor that interferes with lymphedema treatment, thereby also allowing the progression of fibrotic changes. Finally, we did not analyze the various risk factors that could affect changes in fibrosis; these include the type of treatment administered, presence or absence of cellulitis, and lymph node invasion patterns. A prospective study is necessary in the future to address these limitations.

## 5. Conclusions

In summary, inflammation caused by lymph stasis induces fibrosis within the epifascial tissue of the affected extremities. Resolution of lymphedema can halt fibrotic changes in the reversible phase, and this can be confirmed by CT-based quantitative analysis. Future studies are needed to confirm whether the effect of lymphedema treatment on changes in fibrosis depends on the severity of the lymphedema.

## Figures and Tables

**Table 1:** Demographic and clinical data for patients (*n* = 45).

Variables		No. (%)	Mean ± SD
Age (years)			57.82 ± 11.15
Body weight (kg)			60.12 ± 7.13
Height (cm)			156.82 ± 5.84
BMI (kg/m^2^)			24.52 ± 3.54
Duration between surgery and initial CT (months)			54.73 ± 61.15
Duration between initial and follow-up CT (months)			29.00 ± 16.73
Cancer type	Cervical cancer	33 (73)	
	Ovarian cancer	4 (10)	
	Tubal cancer	1 (2)	
	Endometrial cancer	6 (13)	
	Leiomyosarcoma	1 (2)	
Affected side	Right	23 (51)	
	Left	22 (49)	
History of cellulitis		18 (40)	
CTx		21 (46)	
RTx		19 (42)	

CT, Computed tomography. CTx, Chemotherapy. RTx, Radiation therapy.

**Table 2:** Comparison of parameters between affected and unaffected limbs in the initial CT scan.

		Mean ± SD		
		Affected	Unaffected	*p* Value
Circumference (mm)	U20	599.97 ± 11.04	551.36 ± 42.02	0.00 *
	U10	506.34 ± 61.87	452.73 ± 39.65	0.00 *
	L10	397.53 ± 52.62	357.11 ± 31.79	0.00 *
	L20	328.10 ± 45.20	286.02 ± 24.90	0.00 *
Skin & subcutaneous fibrosis (mm^2^)	U20	13,962.25 ± 3922.00	1667.23 ± 537.45	0.00 *
	U10	9327.51 ± 2682.11	1060.43 ± 304.11	0.00 *
	L10	2575.82 ± 2289.67	775.05 ± 235.21	0.00 *
	L20	2363.54 ± 1836.97	720.67 ± 227.54	0.00 *
Fibrosis ratio	U20	27.72 ± 16.80	16.05 ± 4.72	0.00 *
	U10	37.21 ± 21.76	19.76 ± 8.48	0.00 *
	L10	49.35 ± 21.64	31.04 ± 11.27	0.00 *
	L20	55.06 ± 22.53	35.35 ± 12.24	0.00 *

L10, 10 cm below the lower margin of the patellar bone; L20, 20 cm below the lower margin of the patellar bone; U10, 10 cm above the upper margin of the patellar bone; U20, 20 cm above the upper margin of the patellar bone. * *p* < 0.05.

**Table 3:** The difference of circumference, fibrosis, and ratio of fibrosis between the initial and follow-up CT in two study groups.

		Median (Q1, Q3)			
		Total	Improved Group	Aggravated Group	*p* Value
U20	Circumference (mm)	−2.39 (−17.34, 14.41)	−12.55 (−31.35, −6.15)	21.75 (5.8, 44.07)	0.00 *
	S-fibrosis (mm^2^)	55.75 (−485.73, 934.77)	29.00 (−426.41, 420,12)	412.94 (−624.08, 2676.71)	0.29
	Fibrosis ratio	−2.75 (−10.0, 1.92)	−6.40 (−15.7, 1.00)	−0.90 (−6.25, 2.15)	0.00 *
U10	Circumference (mm)	−0.89 (−56.37, 1026.89)	−14.60 (−28.00, −2.85)	12.42 (2.47, 32.55)	0.00 *
	S-fibrosis (mm^2^)	66.25 (−270.56, 656.83)	−228.71 (−693.40, 208.56)	638.53 (21.48, 1520.50)	0.00 *
	Fibrosis ratio	−1.60 (−8.70, 1.90)	−1.60 (−11.2, 0.70)	−2.35 (−6.22, 2.83)	0.24
L10	Circumference (mm)	3.48 (−6.22, 27.88)	−0.02 (−15.23, 12.48)	20.91 (2.97, 37.61)	0.00 *
	S-fibrosis (mm^2^)	219.32 (−122.35, 910.36)	2.38 (−201.63, 435.24)	806.38 (182.19, 1918.05)	0.00 *
	Fibrosis ratio	−4.30 (−10.8, −4.30)	−14.6 (−20.8, −5.40)	−0.80 (−5.35, 1.95)	0.00 *
L20	Circumference (mm)	3.59 (−7.53, 19.05)	−8.90 (−14.96, −2.97)	16.14 (5.17, 32.90)	0.00 *
	S-fibrosis (mm^2^)	263.47 (−56.37, 1026.89)	−71.42 (−947.19, 172.00)	828.47 (263.47, 1620.94)	0.00 *
	Fibrosis ratio	−6.10 (−20.0, −0.10)	−12.2 (−23.5, −5.40)	−2.10 (−4.60, 1.45)	0.00 *

L10, 10 cm below the lower margin of the patellar bone; L20, 20 cm below the lower margin of the patellar bone; S-fibrosis, fibrosis of the skin and subcutaneous tissue; U10, 10 cm above the upper margin of the patellar bone; U20, 20 cm above the upper margin of the patellar bone. The differences in circumference and fibrosis of the skin and subcutaneous tissue between the affected and unaffected sides were used for statistical analysis. The fibrosis ratio on the affected side was used for statistical analysis. * *p* < 0.05.

## Data Availability

Data are available on a reasonable request from the authors.

## References

[1] Kim K., Choi S.C., Ryu S.Y., Kim J.W., Kang S.B. (2008). Major clinical research advances in gynecologic cancer 2008. J. Gynecol. Oncol..

[2] Kang S.H., Lee D.G. (2021). Periclavicular Lymph Node Activation Maintains the Lymphatic Circulation of Upper Extremity Following Breast Cancer Surgery with Axillary Lymph Node Dissection. Lymphat. Res. Biol..

[3] Ridner S., Deng J., Fu M., Radina E., Thiadens S., Weiss J., Dietrich M., Cormier J., Tuppo C., Armer J. (2012). Symptom burden and infection occurrence among individuals with extremity lymphedema. Lymphology.

[4] McDuff S.G., Mina A.I., Brunelle C.L., Salama L., Warren L.E., Abouegylah M., Swaroop M., Skolny M.N., Asdourian M., Gillespie T. (2019). Timing of lymphedema after treatment for breast cancer: When are patients most at risk?. Int. J. Radiat. Oncol. Biol. Phys..

[5] Ly C.L., Kataru R.P., Mehrara B.J. (2017). Inflammatory manifestations of lymphedema. Int. J. Mol. Sci..

[6] Kendall R.T., Feghali-Bostwick C.A. (2014). Fibroblasts in fibrosis: Novel roles and mediators. Front. Pharmacol..

[7] Lynch L.L., Mendez U., Waller A.B., Gillette A.A., Guillory R.J., Goldman J. (2015). Fibrosis worsens chronic lymphedema in rodent tissues. Am. J. Physiol. Heart Circ. Physiol..

[8] Kataru R.P., Baik J.E., Park H.J., Wiser I., Rehal S., Shin J.Y., Mehrara B.J. (2019). Regulation of immune function by the lymphatic system in lymphedema. Front. Immunol..

[9] Wynn T.A., Ramalingam T.R. (2012). Mechanisms of fibrosis: Therapeutic translation for fibrotic disease. Nat. Med..

[10] Azhar S.H., Lim H.Y., Tan B.-K., Angeli V. (2020). The unresolved pathophysiology of lymphedema. Front. Physiol..

[11] Yoo J.S., Chung S.H., Lim M.C., Kim Y.J., Kim K.G., Hwang J.H., Kim Y.H. (2017). Computed tomography-based quantitative assessment of lower extremity lymphedema following treatment for gynecologic cancer. J. Gynecol. Oncol..

[12] Lee D.G., Lee S., Kim K.T. (2022). Computed Tomography-Based Quantitative Analysis of Fibrotic Changes in Skin and Subcutaneous Tissue in Lower Extremity Lymphedema Following Gynecologic Cancer Surgery. Lymphat. Res. Biol..

[13] Schindelin J., Arganda-Carreras I., Frise E., Kaynig V., Longair M., Pietzsch T., Preibisch S., Rueden C., Saalfeld S., Schmid B. (2012). Fiji: An open-source platform for biological-image analysis. Nat. Methods.

[14] Long D.E., Villasante Tezanos A.G., Wise J.N., Kern P.A., Bamman M.M., Peterson C.A., Dennis R.A. (2019). A guide for using NIH Image J for single slice cross-sectional area and composition analysis of the thigh from computed tomography. PLoS ONE.

[15] Tucureanu M.M., Rebleanu D., Constantinescu C.A., Deleanu M., Voicu G., Butoi E., Calin M., Manduteanu I. (2018). Lipopolysaccharide-induced inflammation in monocytes/macrophages is blocked by liposomal delivery of Gi-protein inhibitor. Int. J. Nanomed..

[16] Panos R.J., Mortenson R.L., Niccoli S.A., King T.E. (1990). Clinical deterioration in patients with idiopathic pulmonary fibrosis: Causes and assessment. Am. J. Med..

[17] Van Linthout S., Miteva K., Tschöpe C. (2014). Crosstalk between fibroblasts and inflammatory cells. Cardiovasc. Res..

[18] Mack M. (2018). Inflammation and fibrosis. Matrix Biol..

[19] Sun D., Yu Z., Chen J., Wang L., Han L., Liu N. (2017). The Value of Using a SkinFibroMeter for Diagnosis and Assessment of Secondary Lymphedema and Associated Fibrosis of Lower Limb Skin. Lymphat. Res. Biol..

[20] Taljanovic M.S., Gimber L.H., Becker G.W., Latt L.D., Klauser A.S., Melville D.M., Gao L., Witte R.S. (2017). Shear-wave elastography: Basic physics and musculoskeletal applications. Radiographics.

[21] Polat A.V., Ozturk M., Polat A.K., Karabacak U., Bekci T., Murat N. (2020). Efficacy of Ultrasound and Shear Wave Elastography for the Diagnosis of Breast Cancer-Related Lymphedema. J. Ultrasound Med..

[22] Akita S., Yoshida K., Omura M., Yamaji Y., Tezuka T., Tokumoto H., Azuma K., Ikehara Y., Yamaguchi T., Mitsukawa N. (2021). Noninvasive, objective evaluation of lower extremity lymphedema severity using shear wave elastography: A preliminary study. J. Plast. Reconstr. Aesthet. Surg..

[23] Kim S.Y., Bae H., Ji H.M. (2015). Computed Tomography as an Objective Measurement Tool for Secondary Lymphedema Treated With Extracorporeal Shock Wave Therapy. Ann. Rehabil. Med..

[24] Shin S.U., Lee W., Park E.-A., Shin C.-I., Chung J.W., Park J.H. (2013). Comparison of characteristic CT findings of lymphedema, cellulitis, and generalized edema in lower leg swelling. Int. J. Cardiovasc. Imaging.

[25] Dessources K., Aviki E., Leitao M.M. (2020). Lower extremity lymphedema in patients with gynecologic malignancies. Int. J. Gynecol. Cancer.

[26] Gitas G., Proppe L., Baum S., Kruggel M., Rody A., Tsolakidis D., Zouzoulas D., Laganà A.S., Guenther V., Freytag D. (2021). A risk factor analysis of complications after surgery for vulvar cancer. Arch. Gynecol. Obstet..

[27] Mendez U., Stroup E.M., Lynch L.L., Waller A.B., Goldman J. (2012). A chronic and latent lymphatic insufficiency follows recovery from acute lymphedema in the rat foreleg. Am. J. Physiol. Heart Circ. Physiol..

[28] Mehrara B.J., Park H.J., Kataru R.P., Bromberg J., Coriddi M., Baik J.E., Shin J., Li C., Cavalli M.R., Encarnacion E.M. (2021). Pilot Study of Anti-Th2 Immunotherapy for the Treatment of Breast Cancer-Related Upper Extremity Lymphedema. Biology.

[29] Rockson S.G., Tian W., Jiang X., Kuznetsova T., Haddad F., Zampell J., Mehrara B., Sampson J.P., Roche L., Kim J. (2018). Pilot studies demonstrate the potential benefits of antiinflammatory therapy in human lymphedema. JCI Insight.

[30] Kayıran O., De La Cruz C., Tane K., Soran A. (2017). Lymphedema: From diagnosis to treatment. Turk. J. Surg..

[31] Finnane A., Janda M., Hayes S.C. (2015). Review of the Evidence of Lymphedema Treatment Effect. Am. J. Phys. Med. Rehabil..

[32] Kataru R.P., Wiser I., Baik J.E., Park H.J., Rehal S., Shin J.Y., Mehrara B.J. (2019). Fibrosis and secondary lymphedema: Chicken or egg?. Transl. Res..

[33] Chang D.W., Dayan J., Greene A.K., MacDonald J.K., Masia J., Mehrara B., Neligan P.C., Nguyen D. (2021). Surgical Treatment of Lymphedema: A Systematic Review and Meta-Analysis of Controlled Trials. Results of a Consensus Conference. Plast. Reconstr. Surg..

[34] Brorson H., Ohlin K., Olsson G., Långström G., Wiklund I., Svensson H. (2006). Quality of life following liposuction and conservative treatment of arm lymphedema. Lymphology.

[35] Dionyssiou D., Demiri E., Tsimponis A., Sarafis A., Mpalaris V., Tatsidou G., Arsos G. (2016). A randomized control study of treating secondary stage II breast cancer-related lymphoedema with free lymph node transfer. Breast Cancer Res. Treat..

[36] Koshima I., Inagawa K., Urushibara K., Moriguchi T. (2000). Supermicrosurgical lymphaticovenular anastomosis for the treatment of lymphedema in the upper extremities. J. Reconstr. Microsurg..

[37] Johnstone P.A., Hawkins K., Hood S. (2006). Role of patient adherence in maintenance of results after manipulative therapy for lymphedema. J. Soc. Integr. Oncol..

[38] Brown J.C., Cheville A.L., Tchou J.C., Harris S.R., Schmitz K.H. (2014). Prescription and adherence to lymphedema self-care modalities among women with breast cancer-related lymphedema. Support. Care Cancer—Off. J. Multinatl. Assoc. Support. Care Cancer.

[39] Ergin G., Şahinoğlu E., Karadibak D., Yavuzşen T. (2018). Effect of Bandage Compliance on Upper Extremity Volume in Patients with Breast Cancer-Related Lymphedema. Lymphat. Res. Biol..

